# Characteristics of gut microbiome in patients with pediatric solid tumor

**DOI:** 10.3389/fped.2024.1388673

**Published:** 2024-07-04

**Authors:** Xiaoran Du, Xu Cui, Rongrong Fan, Juntao Pan, Xichun Cui

**Affiliations:** Pediatric Surgery Department, The First Affiliated Hospital of Zhengzhou University, Zhengzhou, China

**Keywords:** gut microbiome, pediatric solid tumor, healthy controls, 16S rRNA sequencing, microbial marker

## Abstract

**Background:**

Pediatric solid tumors are a common malignant disease in children, and more and more studies have proved that there is an inseparable relationship between adult tumors and intestinal microbiome, but the changes in the intestinal microbiota of pediatric solid tumor (PST) patients have been scarcely examined. This study aims to examine the differences in the intestinal microbiota features between patients diagnosed with PST and healthy controls (HCs).

**Methods:**

To elucidate the unique characteristics of the gut microbiota in pediatric patients with solid tumors, we recruited 23 PST patients and 20 HCs. A total of 43 stool samples were gathered, and then 16S rRNA sequencing was performed.

**Results:**

We noticed a noticeable pattern of elevated diversity in the gut microbiota within the PST groups. The differences in microbial communities among two groups were remarkable, regarding the analysis at the class level, the abundance of *Bacilli* was markedly increased in PST patients compared to HCs (*P* < 0.05), regarding the analysis at the genus level, The presence of *Enterococcus* was significantly higher in PST cases compared to HCs (*P* < 0.01), while *Lachnospiraceae unclassified, Lachnospira, Haemophilus* and *Colidextribacter* in PST cases, the abundance was significantly reduced. (*P* < 0.05), 6 genera, including *Bacilli, Lactobacillales, Enterococcaceae* and *Morganella*, showed a significant enrichment compared to healthy controls, while 10 genera, including *Bilophila, Colidextribacter, Pasteurellales, Haemophilus, Lachnospiraceae unclassified, Lachnospira* and *Fusobacteriales*, were significant reduction in the PST groups.

**Conclusion:**

Our research conducted the characterization analysis of the gut microbiota in PST patients for the first time. More importantly, there are some notable differences in the gut microbiota between PST patients and healthy controls, which we believe is an interesting finding.

## Introduction

Childhood malignancies have been a gradual increase in the incidence over the past three decades, and the incidence has been rising at an annual rate of about 1% when considering all types of cancer combined (1). This translates to approximately 400,000 cases per year globally (2), and standing as the second leading cause of mortality in developed countries after accidents (3). There are numerous types of PST, with wilms tumor, osteosarcoma, and neuroblastoma being the most common types of childhood cancers (4). Neuroblastoma, constituting roughly 15% of all cancer-related fatalities, stands as the prevalent solid tumor among infants (5). Osteosarcoma stands as the predominant primary malignant bone tumor among the demographic of children and adolescents (6). Wilms tumor prevails as the primary kidney tumor in childhood, constituting 90% of occurrences (7). The occurrence of PST is linked to early developmental processes (8). Considering their emergence within actively growing tissues, childhood solid tumors can be viewed as diseases arising from dysregulated development (9). PST demonstrates high metastatic potential, poor prognosis, and substantial resistance to current therapeutic methods (10). Cancer continues to be a prominent contributor to mortality in the pediatric and adolescent populations (11). Each year, around 15,000 children and adolescents aged 0–19 receive a diagnosis of cancer in the United States (12). It is crucial for primary care physicians and parents to have a thorough understanding of the initial symptoms of childhood malignancies to enable effective early detection and treatment (13). When it comes to diagnosis, creating tests for children has proven to be more challenging (14).

The human microbiota encompasses all microorganisms, such as bacteria, viruses, fungi, archaea, and protozoa, residing within the human body (15). The primary habitat of microorganisms is the gut, but a plethora of microorganisms can also be found in various parts of the human body, including digestive system, skin, reproductive system and respiratory system (16). In human bodys, there is no denying that the largest microbial ecosystem is the gut microbiota (17). Recently, due to the progress in gut microbiome study, there has been an increasing acknowledgment of the intimate link between gut microbes and diseases, such as nonalcoholic fatty liver (18), obesity (19), heart failure (20), diabetes (21) and depression (22). Meanwhile, a growing body of research suggests an inseparable relationship between the occurrence and development of adult tumors and the gut microbiota. For instance, resident gut bacteria can influence patients' response to cancer immunotherapy (23), the gut microbiota possesses a unique ability to influence the tumor microenvironment as well as the metabolism of chemotherapy drugs or medications (24), the gut microbiota can modulate melanoma patients' response to PD-1 immunotherapy (25), the gut microbiota and its metabolites change with different stages of lung cancer development (26), the composition of the gut microbiota in patients diagnosed with colorectal cancer differs significantly from that of healthy individuals (27), based on specific changes in the gut microbiota, it is possible to screen for gut microbiota-targeted biomarkers and establish a diagnostic model, potentially serving as a non-invasive tool for distinguishing hepatocellular carcinoma (28), However, many aspects, including the mechanisms of onset, age of onset and characteristic features, differ between pediatric tumors and adult tumors. For instance, compared to adult tumors, childhood tumors generally exhibit a lower overall mutation burden (9). Similarly, genomic sequencing research has highlighted distinctions between adult and pediatric tumors. Unlike many adult tumors, which exhibit significant somatic mutations, pediatric tumors generally feature a limited number of somatic mutations in their cells (29). Therefore, we cannot indiscriminately merge the diagnosis and treatment of PST with those of adult tumors.

Over the recent decades, there has been an increasing enthusiasm for exploring the human microbiome and its implications for both well-being and illness. The primary emphasis of these investigations has revolved around understanding the changes in microbial community composition across various circumstances (30). However, research on the gut microbiome of PST patients has been limited thus far. This is indeed the purpose behind our undertaking of this research. This research explores the gut microbiome attributes in pediatric patients with solid tumors in the central region of China. We conducted analysis of fecal samples from 20 HCs and 23 PST patients using the 16S rRNA MiSeq sequencing method. The aim was to investigate the distinctive composition of gut microbiota in PST patients and observe the specific changes of intestinal microecology in children with solid tumor.

## Materials and methods

### Participant information

This research was rigorously planned and executed in accordance with the PRoBE guidelines (Prospective Specimen Collection and Retrospective Blinded Evaluation), in adherence to the principles set forth in the Helsinki Declaration and the Regulations of Good Clinical Practice. This research collected fecal samples for analysis and all samples from the First Affiliated Hospital of Zhengzhou University. Every participant was newly diagnosed with PST. The participants in this research were identified as individuals diagnosed with PST through pathological examination and tissue biopsy. The participants exclusively comprised patients who were newly diagnosed with PST. The exclusion criteria are as follows: (1) Simultaneous existence of additional illnesses, (2) Administration of antibiotics in the last eight weeks, and (3) medical history involves past treatments or tumor removal surgery such as chemotherapy and radiotherapy. There were 50 fecal samples were gathered in the scope of this research. These included 25 samples from patients diagnosed with PST and 25 samples from HCs. After thorough screening, 20 HCs and 23 PST patients were selected for the study, ensuring matching criteria for sex, age and BMI. In the end, 16S rRNA MiSeq sequencing was performed on 43 fecal samples were collected from both PST and HCs.

Clinicopathological and demographic information of participants was obtained through both electronic medical records and questionnaires from the hospital (Table 1).

**Table 1:** Demographic characteristics of patients with pediatric solid tumor and the age- and gender-matched healthy controls.

Characteristics		PST	HC
*N*		23	20
Gender	Male	14 (60.9%)	20 (100%)
	Female	9 (39.1%)	0
Age(years)		5.1 [1.0, 7.0]	5.1 [2.0, 11.0]
BMI(kg/m^2^)		18.7 [14.6,21.8]	16.7 [14.8,20.9]
Disease type	Sarcoma	4 (17.4%)	–
	Neurogenic tumor	7 (30.4%)	–
	Teratoma	5 (21.8%)	–
	Other tumors	7 (30.4%)	–
History of surgery		0	0
Any other comorbidities		0	0
Use of antibiotics		0	0
Radiotherapy or chemotherapy		0	0

Data are presented as a number with the percentage in parentheses, or as the median with the interquartile range (IQR) in square brackets. PST, pediatric solid tumor; HCs, healthy controls.

### Collection of human fecal samples

Participants provided a new stool specimen for the research. Standard stool examinations were conducted to evaluate the texture of the stool. The specimens were split into five portions, each weighing 200 mg, and immediately stored at −80°C.

### DNA extraction and PCR amplification

Consistent techniques were employed across each sample, and the procedures were consistently executed by laboratory staff members. The specimen was blended with 790 μl of a sterile lysis buffer, this mixture was then placed in a 2 ml screw-cap tube containing 1 g glass beads(0.1 mm BioSpec Products, Inc., USA). The blend was thoroughly vortexed and subsequently subjected to incubation at 70°C for one hour. Afterward, the combination underwent bead beating at the highest speed setting for 10 min. Using The E.Z.N.A.® Stool DNA Kit (Omega Bio-tek, Inc., GA), Bacterial DNA extraction was carried out in accordance with the instructions furnished by the producer. The isolated DNA was preserved at −20°C. Amplification of the 16S rRNA genes was performed targeting the V3–V4 region using the DNA extracted from individual samples as a template. In each sample, PCR was utilized to amplify the V3–V4 region, employing primers R2 and F1 with sequences 5′-CCTACGGGNGGCWGCAG-3′ and 5′-GACTACHVGGGTATCTAATCC-3′, respectively. Following the specified protocol. PCR reactions were performed utilizing the EasyCycler 96 PCR system provided by Analytik Jena Corp., AG.

### Miseq sequencing and sequence data processing

The amplified products from different samples were tagged with unique indexes and combined in equal proportions to enable sequencing. The sequencing procedure was carried out on the Miseq platform (Illumina Inc., USA) by Shanghai Mobio Biomedical Technology Co. Ltd., adhering to the manufacturer's guidelines. After arranging the chosen readings, they were categorized into specific sample groups using dedicated barcodes. Following that, the primers and barcodes were eliminated.

Utilizing FLASH v. 1.2.10 to combine paired-end sequences from individual libraries. Utilizing default parameters to enable the merging of sequences with overlapping regions (31). The merged reads obtained from the FLASH procedure underwent an evaluation of quality. Utilizing UCHIME version 4.2.40 to detect and remove potential chimera sequences (32). All nucleotide sequences from the samples were deposited into the NCBI database(National Center for Biotechnology Information) in the United States under the project accession number PRJNA1026296.

### OTU clustering and taxonomic annotation

The same quantity of reads was randomly chosen from each of the samples. Following this, The UPARSE pipeline was employed for operational taxonomic unit (OTU) classification (33). We computed the total count of OTUs (operational taxonomic units) across different taxonomic levels, such as genus, family, order, class and phylum (34).

### Bacterial diversity and taxonomic analysis

Utilizing the “vegan” R package, we calculated the Shannon index and Simpson index to assess bacterial community diversity. Moreover, ace estimators were employed to estimate the abundance of the bacterial community. Using Venn diagrams to illustrate the similarity and overlap of OTUs, showing common and distinct units present in different samples. Additionally, the creation of heatmaps to visually represent dominant species in the dataset was facilitated by the utilization of Heatmap Builder. We generated bar plots to illustrate the microbial community by analyzing the species composition. Utilized for performing Principal Coordinates Analysis (PCoA) and Non-Metric Multidimensional Scaling (NMDS) was the “vegan” R package, and the objective was to clarify the microbial differences among the samples. To aid in identification, used for computing both weighted and unweighted UniFrac distances was the “phyloseq” package. The evolutionary relationships among bacteria were visualized through phylogenetic trees. Bacterial taxonomic analyses were conducted at various levels, including genus, family, order, class and phylum. Afterward, we employed Wilcoxon rank-sum tests to assess microbiome differences among two groups. Furthermore, applying the LEfSe (linear discriminant analysis effect size) method, we conducted LDA (linear discriminant analysis) to identify critical microbiomes that exhibit significant distinctions. Utilizing the LEfSe online tool accessible at http://huttenhower.sph.harvard.edu/lefse/e/, the analysis was performed. An LDA score threshold of log10 = 2 was set (35). Additionally, using both the Wilcoxon rank-sum test and the nonparametric Kruskal-Wallis rank-sum test to identify significant biomarkers.

### Statistical analysis

Utilizing IBM SPSS Statistics version 20.0 (IBM Corp., Armonk, NY, USA), the data analysis was conducted. Statistical significance of differences between the groups was determined. Spearman's rank test was applied for correlation analysis. To compare categorical variables, Fisher's exact test was utilized, while for continuous variables, the Wilcoxon rank-sum test was utilized.

## Results

### Participant profiles

**Figure 1:**
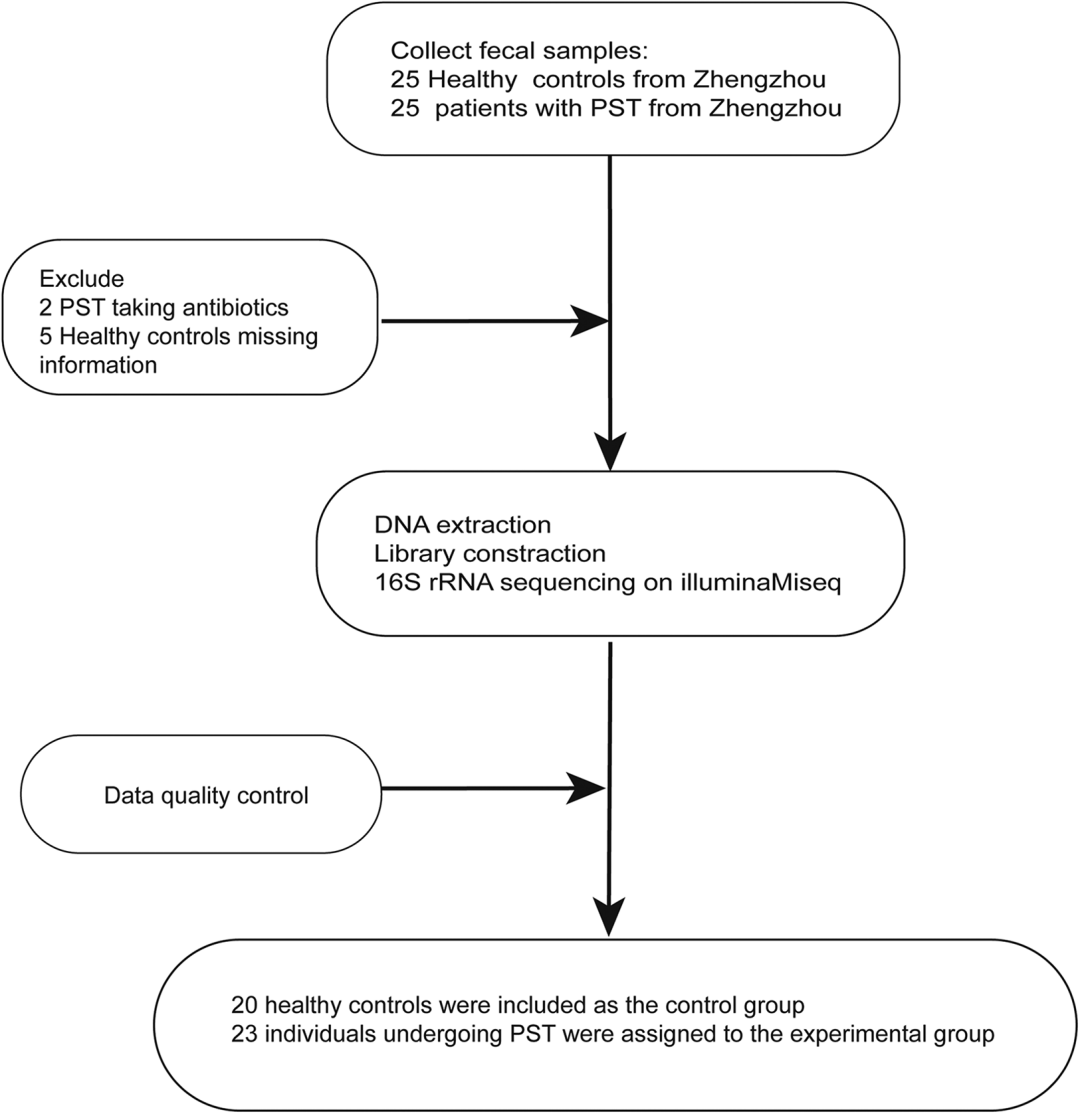
Study design and flow diagram. A total of 50 fecal samples from Central China were collected. After a strict pathological diagnosis and exclusion process, 23 PST patients and 20 HCs were included, 20 HCs were included as the control group, 23 individuals undergoing PST were assigned to the experimental group. PST, pediatric solid tumor; HCs, healthy controls.

The study collected 50 stool samples, including 25 PST and 25 HCs. After rigorous screening and exclusion (Figure 1), 43 stool samples remained, comprising 23 PST patients and 20 HCs. Ultimately, The fecal microbiota composition of 20 HCs and 23 PST patients was analyzed.

### The increased gut microbial diversity in PST

**Figure 2:**
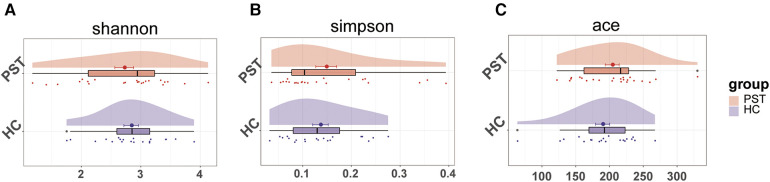
The microbial diversity was increased in PST patients (*N* = 23) vs. HCs (*N* = 20). As measured by the Shannon (**A**) and Simpson (**B**) index, the intestinal microbiota diversity in PST patients was significantly higher than in HCs. This finding was confirmed by the ace indices (**C**) PST, pediatric solid tumors; HCs, healthy controls.

A marked increase in gut microbial diversity in PST patients compared to HCs. As measured by the Shannon (Figure 2A) and Simpson (Figure 2B) indices, there was a notable increase in the diversity of intestinal microbiota in PST patients. The observed and ace indices confirmed the validity of this finding (Figure 2C).

### Disparities in the gut microbiome between HCs and PST patients

**Figure 3:**
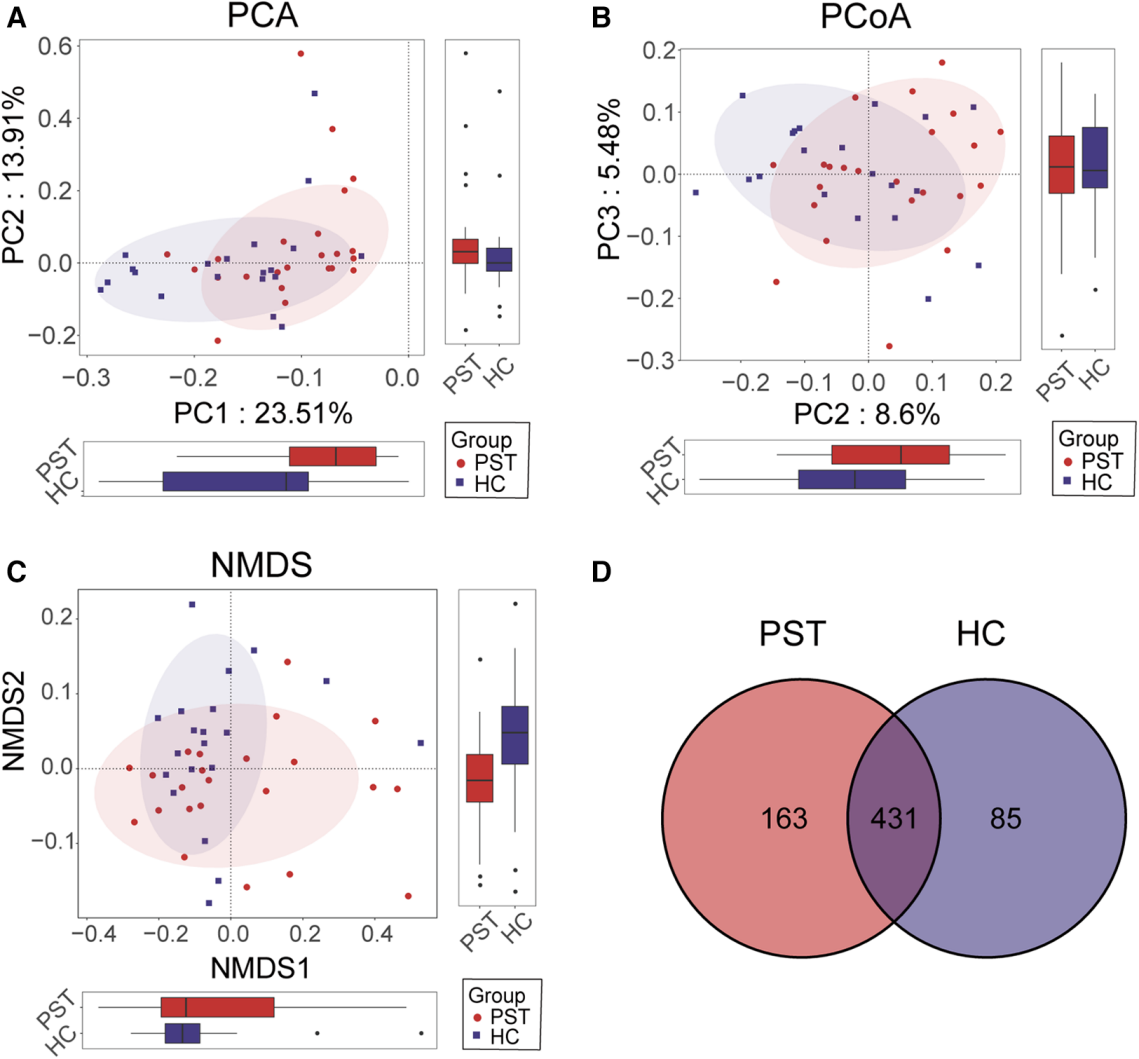
Comparison of beta diversity between PST patients (*N* = 23) and HCs (*N* = 20). According to PCA (**A**), PCOA (**B**) and NMDS (**C**) analysis, there were significant differences in OTU distribution between PST and HCs. The Venn diagram (**D**) revealed that out of 679 OTUs, 431 OTUs were shared between the two groups, while 163 OTUs were specific to PST. PST, pediatric solid tumor; HCs, healthy controls; OTUs, operational taxonomic units; NMDS, nonmetric multidimensional scaling; PCA, principal component analysis; PCoA, principal coordinate analysis.

Furthermore, beta diversity analysis was conducted to illustrate the differences in microbial community composition among individual samples. PCA (Figure 3A), PCoA (Figure 3B) and NMDS (Figure 3C) analyses estimated significant differences in OTU distribution between the two groups. Figure 3D of the Venn diagram illustrated the sharing of 431 out of 679 OTUs between the PST and HCs. Notably, among these 679 OTUs, 163 were unique to the PST group.

### Clustering of operational taxonomic units (OTUs) and taxonomic analysis

**Figure 4:**
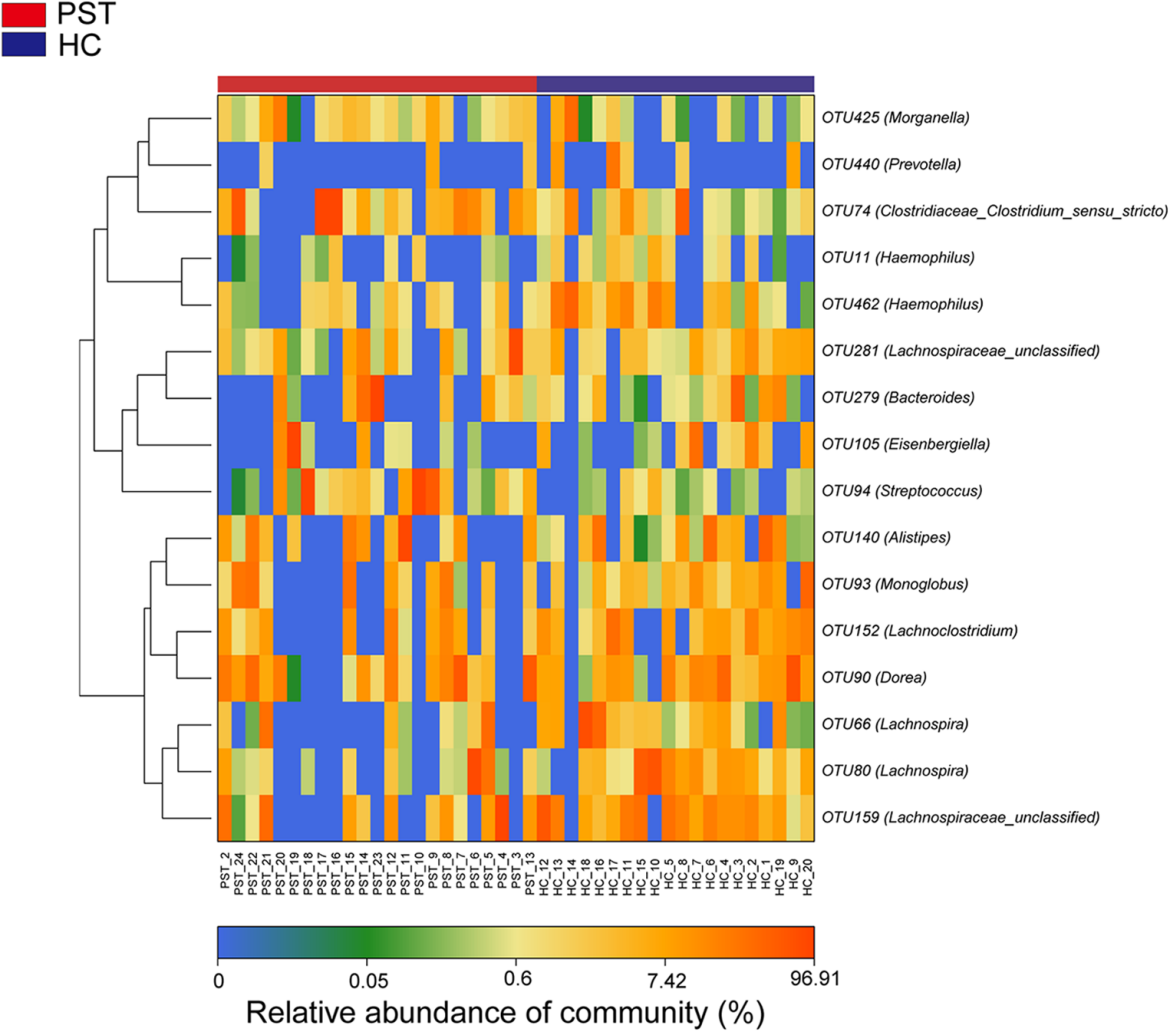
Heatmap displayed the relative abundance differences of OTUs between PST (*N* = 23) and HCs (*N* = 20). The relative abundance of each sample was shown, with red indicating high abundance and blue indicating low abundance. Each row represents an OTU. OTU, operational taxonomic unit; PST, pediatric solid tumors; HCs, healthy controls.

The heatmap illustrates the variation in relative abundance of OTUs between PST and HCs (Figure 4). Shades of blue are used to represent OTUs with lower relative abundances, whereas shades of red indicate higher relative abundances. A total of 8 bacterial species show an increase in PST patients, including OTU94 (Streptococcus), OTU74 (Clostridiaceae Clostridium sensu stricto), OTU279 (Bacteroides), OTU93 (Monoglobus), OTU140 (Alistipes), OTU425 (Morganella), OTU281 (Lachnospiraceae unclassified) and OTU105 (Eisenbergiella).

### Composition and comparison of the gut microbiome in PST and HCs

**Figure 5:**
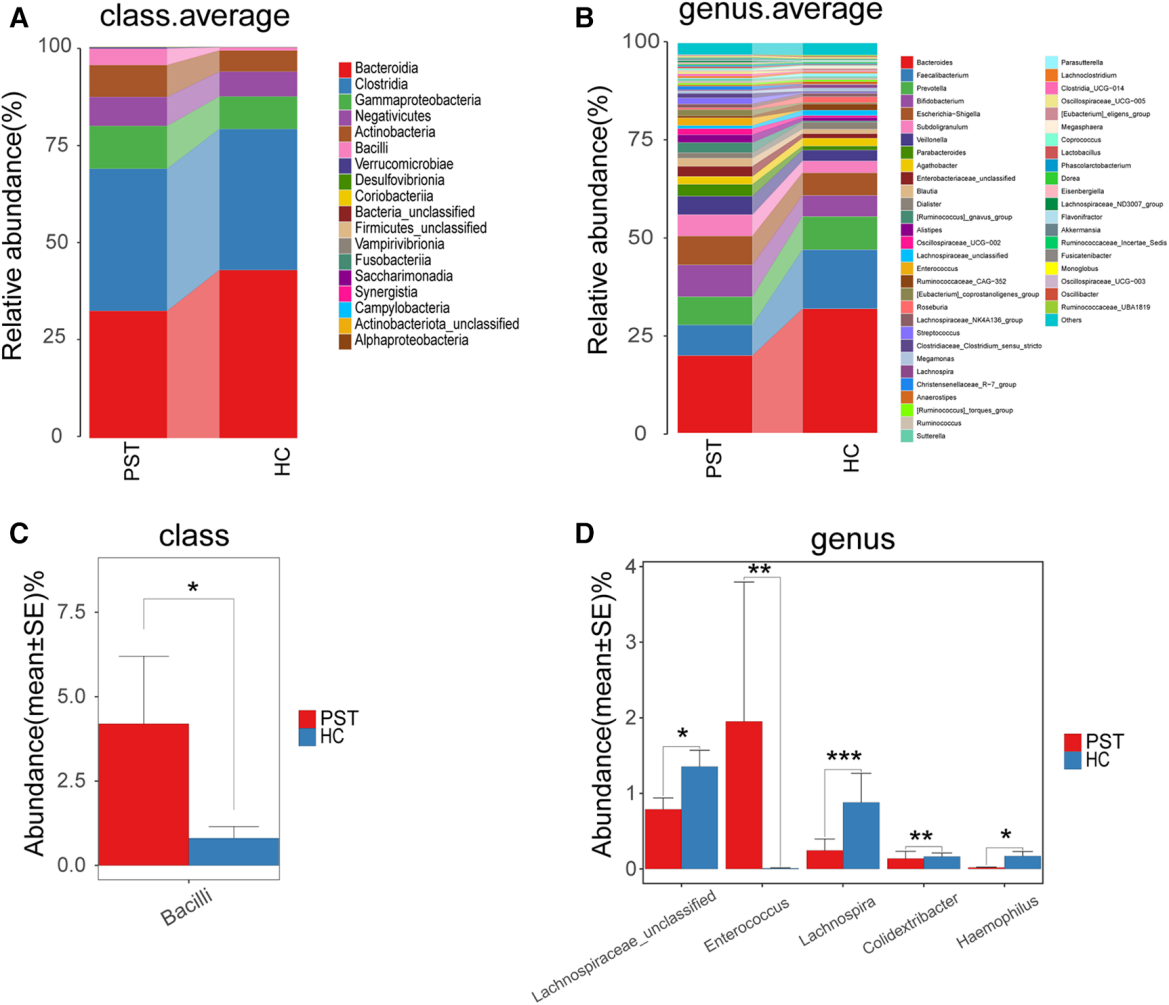
Composition and comparison of the gut microbiome in PST patients (*N* = 23) and HCs (*N* = 20). The class level (**A**) and genus level (**B**) composition diagrams showed the composition characteristics of the two groups of gut microbiome. The differences in the relative abundance of key bacterias in the two groups were compared at the class level (**C**) and the genus level (**D**) The relative abundance of each bacteria was represented by the mean ± SE. We used the Wilcoxon rank-sum test to evaluate whether the difference of relative abundance was significant (*P < 0.05; **P < 0.01 and ***P < 0.001). PST, pediatric solid tumor; HCs, healthy controls.

The gut microbiota of PST and HCs were analyzed and compared in terms of their composition and diversity. The computed relative prevalence of individual sample was visually depicted across diverse taxonomic tiers through the utilization of OTU annotations. At the level of class, there was a predominant average proportion observed in both cohorts (nearly 99%) of *Bacteroidia, Clostridia, Gammaproteobacteria, Negativicutes, Actinobacteria* and *Bacilli* (Figure 5A). Similarly, at the genera level, including *Faecalibacterium, Prevotella, Bacteroides, Bifidobacterium, Subdoligranulum, Escherichia-Shigella, Veillonella* and *Parabacteroides*, both groups exhibited a consistent pattern where an average of 90% of the microbiota consisted of these 29 genera (Figure 5B). Regarding the classifications of class and genus, notable differences were observed between the two groups in microbial composition.

Subsequently, the gut microbiota of 23 PST and 20 HCs were compared at every taxonomy level. We utilized Wilcoxon rank-sum tests to examine substantial disparities between PST and HCs in microbial composition. In the analysis at the class level, the abundance of *Bacilli* was markedly elevated in PST in comparison to HCs (*P* < 0.05) (Figure 5C). In the analysis at the genus level, the presence of *Enterococcus* showed a significant elevation in PST in comparison to HCs (*P* < 0.01). While *Lachnospiraceae unclassified, Lachnospira, Colidextribacter* and *Haemophilus* significantly decreased (*P* < 0.05) (Figure 5D). In analyses conducted at both the class and genus levels, significant differences were observed.

### Phylogenetic characteristics of gut microbial communities in PST

Utilizing OTU-based LDA scores and Lefse analysis, 6 genera exhibited notable enrichment in PST. Furthermore, 10 genera have been identified as predominant in the HCs, noteworthy disparities were identified among groups.

**Figure 6:**
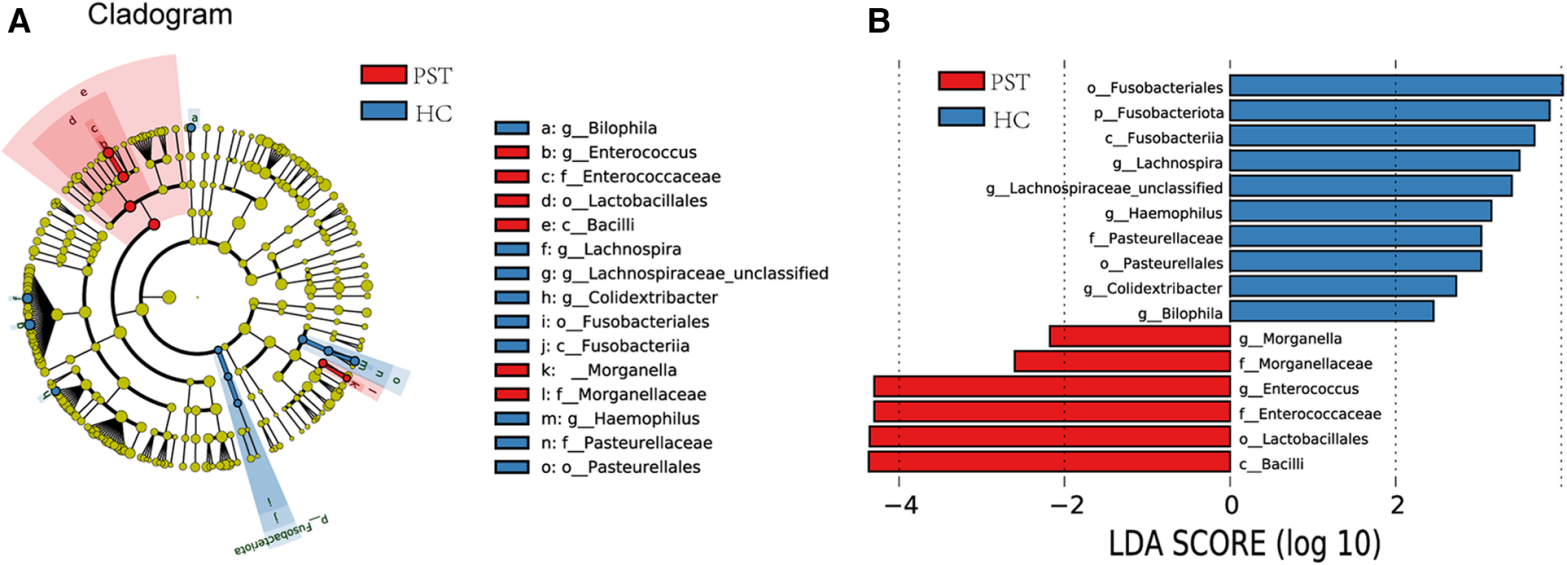
Lefse and LDA analysis based on OTUs characterize the microbiome between PST and HCs. The phylogenetic tree (**A**) diagram by the LEfSe method showed the phylogenetic distribution of the gut microbiome related to PST and HCs. The circles radiating from the inside to the outside represented the classification level from the phylum to the genus. Each circle at levels represented a classification at that level, and the diameter of the circle represents its relative abundance. The uniform coloring with no significant difference was yellow, and the biomarker with significant difference followed the grouping color for coloring. Species with an LDA score greater than 2 and a *P*-value less than 0.05 were considered different species. LDA score histogram (**B**) showed the gut microbiome with significant differences between the two groups. The higher the LDA score, the higher the importance of microbial biomarkers. The default LDA score was greater than 2, and the *P*-value was less than 0.05, which was considered to indicate differential species. LDA, linear discriminant analysis; LEfSe, linear discriminant analysis effect size; OTUs, operational taxonomic units; PST, pediatric solid tumor; HCs, healthy controls.

Figure 6A visually demonstrates the phylogenetic distribution of gut microbial communities between two groups. In Figure 6B, histograms illustrating LDA scores for selected taxonomic clusters are presented, indicating bacteria exhibited notable differences between two groups.

Correspondingly, 6 biomarkers, including *Bacilli, Lactobacillales, Enterococcaceae, Enterococcus, Morganellaceae* and *Morganella*, exhibited significant enrichment in PST (*P* < 0.05), affirming their position as predominant genera. Moreover, there was a notable decrease in the prevalence of 10 genera, including *Bilophila, Colidextribacter, Pasteurellales, Haemophilus, Lachnospiraceae unclassified, Lachnospira* and *Fusobacteriales* (*P* < 0.05).

## Discussion

This research provides the first comprehensive analysis of gut microbiota alterations in pediatric solid tumor patients, including elements of both community composition and species diversity. Sequencing of the gut microbiota was conducted using samples collected from 23 PST and 20 HCs matched for age, sex, and BMI, all from the central region of China, using the 16S rRNA gene. Surprisingly, PST patients exhibited a substantial increase in gut microbiota diversity in comparison to the HCs. In contrast, patients diagnosed with cirrhosis and liver cancer show a decrease in microbial diversity (36). The precise factors contributing to the diversity variation among gut microbial communities remain unknown (37). Disease occurrence might be linked to changes in microbial diversity, with increases or decreases that can vary based on different diseases. For example, in individuals diagnosed with AIH (autoimmune hepatitis), there was a marked elevation in *Veillonella*, coupled with a concurrent reduction in *Streptococcus*, as opposed to HCs (38). Within the high AAC (Acute Pancreatitis) score group, there was an observed trend of decreasing microbial diversity compared to HCs (39). The group with chronic pancreatitis showed lower microbial diversity compared to the healthy controls (40). Patients diagnosed with breast cancer exhibited reduced diversity in their intestinal microbiota in comparison to HCs (41). NMDS PCA and PCoA analyses revealed notable disparities when comparing PST patients and HCs in the composition of the gut microbiota community. The utilization of LefSe analysis and the resulting LDA scores revealed significant disparities in the gut microbiota composition among two cohorts. There is undoubtedly a connection between changes in microbial diversity and the initiation and advancement of diseases, and this is precisely the purpose of conducting this research.

In this study, 6 biomarkers, including Bacilli, Lactobacillales, Enterococcaceae, Enterococcus, Morganellaceae and Morganella, exhibited significant enrichment in PST (*P* < 0.05), affirming their position as predominant genera. Research suggests that Bacilli bacteria may be associated with the occurrence of colorectal tumors, with bacteria such as Bacillus cereus and Bacillus subtilis found in the intestines of patients with colon cancer (42, 43). Additionally, Bacilli bacteria have potential roles in immune regulation and tumor therapy, including their influence in the tumor microenvironment and their application as immune modulators (25). Some bacteria in the Enterococcaceae family, such as Enterococcus faecalis, are commonly found in the human intestinal tract and can cause infections (44). Some studies have found an association between certain gastrointestinal diseases, such as inflammatory bowel disease, and an increase in the abundance of Enterococcaceae bacteria (45). Enterococcus and Morganella may lead to urinary tract infections and wound infections in immunocompromised or hospitalized patients (46).

As metagenomics research progresses, our comprehension of the gut microbiota has grown, elucidating their increasingly evident connection with various diseases. For instance, gut microbes influence physiology by partially breaking down dietary components and substances produced by the host, leading to the production of bioactive compounds, including toxins (47). Gut microorganisms have a central role in regulating the overall purine balance and serum uric acid (UA) levels in the host (48). The gut microbiota facilitates the anti-obesity effects of intermittent fasting by impeding the absorption of intestinal lipids (49). Differences were noted in the lipid metabolites and gut microbiome when comparing patients with Spinal Muscular Atrophy to the control subjects. The altered microbiota could potentially be associated with the disruption of lipid metabolism in individuals with Spinal Muscular Atrophy (50). Patients with hepatic encephalopathy (HE) and cirrhosis showed reduced richness and diversity of microbial species (51). Moreover, the gut microbiota holds promise for being employed as a diagnostic instrument in clinical settings. The fecal microbiome can distinguish autoimmune hepatitis patients from healthy individuals (52). The gut microbiome can distinguish epilepsy patients from healthy individuals (53). By constructing the gut metagenome, type 2 diabetes can be distinguished from the control group based on its markers. Compared to similar analyses based on human genome variations, the gut microbiome demonstrates higher specificity (54). A research has unveiled complex changes in microbial communities linked to Crohn's disease and identified microbial genes as consistent diagnostic markers applicable across diverse cohorts, including those from different cultural backgrounds and geographical (55). When it comes to the diagnosis of gastrointestinal disorders, the gut microbiome has been acknowledged as a standalone diagnostic tool. Furthermore, strategies to enhance effectiveness across diverse cohorts have been revealed by identifying factors that consistently contribute to alterations in the gut microbiome across various study groups (56). Alterations in gut microbiota composition are associated with colorectal cancer and its precancerous lesions, with increased abundance of Fusobacterium and other bacteria. These microbial characteristics can serve as biomarkers for detecting colorectal tumors (57–59). We hypothesize that detecting gut microbiota could become a new and effective method for differentiating between patients with tumors and those without. It presents benefits including minimal invasiveness, diagnostic efficiency, high acceptability and cost-effectiveness. Therefore, we consider this research to be exceptionally valuable and meaningful. Furthermore, the gut microbiota demonstrates immense potential in the treatment of diseases. For example, scientists are dedicated to addressing inflammatory bowel diseases through the restoration of intestinal microbial balance and the improvement of intestinal inflammation. Novel approaches focused on the gut microbiome, including probiotics, prebiotics, and synbiotics, are emerging as promising methods to decelerate the advancement of inflammatory bowel diseases and promote intestinal health (60). The gut microbiota serves as a potential biomarker for the diagnosis and prognosis of systemic lupus erythematosus, and it also holds promise as a potential target for the treatment of systemic lupus erythematosus (61). Modulating the gut microbiota regulates distal symmetric polyneuropathy in diabetic patients (21). Although there is limited research on the role of the gut microbiota in disease treatment currently, we firmly believe that in the near future, the gut microbiota will play a more significant role in the diagnosis and treatment of diseases, helping a larger number of people. This is the original intention behind our undertaking of this study.

## Conclusion

This research offers the initial assessment of gut microbiota in PST patients. Crucially, we observed significant differences in gut microbes between the two groups, particularly, changes in bacteria such as Bacilli, Lactobacillales, Enterococcaceae, Enterococcus, Morganellaceae, and Morganella. We think this is an interesting finding. In the next step of our experiments, we will increase sample collection and begin constructing relevant diagnostic models based on their specific microbial changes.

### Limitations of study

We acknowledge the limitations of this study. The sample size of the study was relatively small; it is indeed challenging to collect cases of pediatric solid tumors. We spent nearly two years gathering these cases. Many children presented with fever upon admission and were immediately treated with antibiotics, leading to their exclusion from the study. Additionally, some cases involved children who were readmitted due to relapses or post-surgery chemotherapy, and they were also excluded. Consequently, the sample size for this experiment was relatively limited.

This experiment serves as a preliminary study for the comprehensive investigation of gut microbiota changes in pediatric solid tumor patients in the next step. The aim is to observe whether there are differences in the composition of gut microbiota between pediatric solid tumor patients and the healthy control group, facilitating the subsequent research. The results are clear: there are significant differences in the microbiota composition between the two groups. This finding lays the groundwork for our next experiment.

## Data Availability

The datasets presented in this study can be found in online repositories. The names of the repository/repositories and accession number(s) can be found in the article/Supplementary Material.
